# Law, neuroscience, and authenticity by design: protecting users’ minds in the digital sphere

**DOI:** 10.3389/fpsyg.2026.1815392

**Published:** 2026-04-17

**Authors:** Cristina Elena Popa Tache, Catalin Silviu Sararu

**Affiliations:** 1 Centre International de Recherches et Études Transdisciplinaires (CIRET), Paris, France; 2 Danubius International University, Galați, Romania; 3 Lauterpacht Centre for International Law, University of Cambridge, Cambridge, United Kingdom; 4 Academy of Romanian Scientists, Bucharest, Romania; 5 Faculty of Law, Bucharest University of Economic Studies, Bucharest, Romania

**Keywords:** algorithmic governance, Authenticity-by-Design, cognitive autonomy, human rights, mental integrity, persuasive design, transdisciplinary methodology

## Abstract

Contemporary digital technologies reshape human deliberation through micro-targeting, persuasive design, and algorithmic systems capable of anticipating and steering behaviour. Cognitive autonomy thus emerges as a central legal value. The analysis of the European normative framework, correlated with recent literature in neuroscience and behavioural economics, highlights the need to recognise mental integrity as an autonomous object of legal protection. Existing regulations structure data protection, digital competition, and commercial practices, while the dynamics of willformation call for distinct regulatory articulation. The article advances a transdisciplinary vision grounded in legal analysis, scientific knowledge, and technological design within a unified conceptual framework. The concept of Authenticity-by-Design is formulated as an operational model oriented toward cognitive impact assessments, standards of neutral design, and mechanisms of algorithmic transparency. Mental autonomy is conceptualised as an expression of dignity and a structural condition of freedom in an algorithmic society.

## 1 Introduction

In the Digital Milieu, ubiquitous information technologies are introducing new risks relevant to the cognitive autonomy of users. Sophisticated algorithms, micro-targeting on social networks, and artificial intelligence personalise content in ways that can influence people’s decisions and thinking without their awareness. As early as 2019, the Council of Europe warned of the danger that algorithmic processes could determine citizens’ social and political behaviour and prevent them from forming their own opinions independently (Council of Europe, 2019). Looking at this from an inter- and multi- disciplinary angle, this paper asks: how can we protect the user’s ‘soul’/mind in the digital space, and how can an *Authenticity-by-Design* framework guarantee respect for mental autonomy?

In different legal systems or fields (European Union and international), behavioural neuroscience, behavioural economics and technological ethics, we have identified the main gaps in current regulations. We find that European legislation focuses mainly on data protection and e-commerce, without explicitly addressing the protection of cognitive processes or mental integrity (European Commission, n.d.). The General Data Protection Regulation (GDPR) regulates the confidentiality of personal data, including sensitive health data, but does not directly regulate scenarios in which digital interfaces manipulate choices through decision-making. Furthermore, acts that explicitly prohibit dark *patterns* (manipulative design patterns) are fragmented between consumer law, online services [European Data Protection Board (EDPB), 2023] and the Artificial Intelligence Act (Car and Cassetti, 2025).

Based on these findings, we propose an Authenticity-by-Design operational regime to support legislation and technological design toward cognitive autonomy. The reference principles relate to algorithmic transparency, respect for mental privacy, automatic attribution of truthfulness in interfaces, and effective informed consent mechanisms. Technically, we recommend the introduction of mandatory cognitive impact assessments similar to DPIA (Data Protection Impact Assessment, Regulation (EU) 2016/679, 2016) for data and neutral design standards. Politically, we propose amendments to Regulation (EU) 2016/679 (General Data Protection Regulation) and Regulation (EU) 2022/2065 on a Single Market for Digital Services (Digital Services Act) and new regulations (Codes of Good Practice for Digital Design) that impose default measures to protect the mind, such as easily accessible opt-out options and penalties for subtle manipulation. The article also presents a comparative table of existing legal instruments versus key dimensions of cognitive autonomy and an example of a Mermaid chart illustrating the steps of implementing the Authenticity-by-Design programme from defining principles to monitoring.

Protecting cognitive autonomy must not be neglected when we want democracy and dignity, and this requires an integrated approach, combining legislative intervention with good design practices and public education. Although there are some difficulties in clearly defining mental integrity, the rapid pace of technology (passing problems), and measuring cognitive impact, this paper identifies current gaps and provides concrete and operational recommendations. The development of the best possible Authenticity-by-Design framework will be able to guide both legislators and the technology industry toward digital technologies that respect freedom of thought and individual decision-making autonomy (Bertoni and Ienca, 2024, Section 2, para. 2, Regulation (EU) 2024/1689, 2024, Art. 5).

On the other hand, the concept of cognitive liberty is gaining increasing academic and political attention (Pillay, 2025). This refers to the right of individuals to control their own mental processes and thoughts. Cognitive liberty is the right to be free from external interference, invasion, manipulation, or monitoring of the brain’s processes without consent, specifically in regards to biotechnological means. It is not just the right to believe what one chooses, but the right to have control over one’s own cognitive processes (Wolpe, 2017, Chap. 14). Studies in neurotechnology show that neural data can reveal mental states, emotions, or intentions (using electroencephalograms, fMRI signals), and Brain-Computer Interface (BCI) devices even allow the decoding of elements of thought (Szoszkiewicz and Yuste, 2025). For these reasons, technological ethics researchers propose new neurorights, i.e., rights, such as mental integrity, identity and, at their core, cognitive freedom as the right to think without surveillance or manipulation. McCarthy-Jones emphasises that the loss of ‘sovereignty of the mind’ is equivalent to the loss of dignity and democracy, and that freedom of thought must be protected in the digital age (McCarthy-Jones, 2019).

The GDPR protects personal data, including health data, while the DSA and Digital Services Act (DMA) (Regulation – EU – 2022/1925, 2022) prohibits certain forms of manipulation, such as dark patterns on large platforms, and the new AI legislation (AI Act) will prohibit subliminal influencing techniques. However, no legal act clearly enshrines the right to mental integrity or psychologically unbiased decisions (Car and Cassetti, 2025). There is a lack of common definitions and regulatory tools to regulate the subtle cognitive effects of digital interfaces. Therefore, the study’s question is how can law and technological design work together to protect humans in the digital space? The purpose of the article is to critically evaluate the interdisciplinary literature and relevant regulations and to propose a conceptual and practical Authenticity-by-Design system along with legislative principles and recommendations. In the following section, we describe the methodology of the analysis, then summarise the data and gaps identified in the Results, concluding with discussions on the implications and future directions useful in substantiating a public policy adapted to protect the cognitive autonomy of digital users.

### 1.1 Methods

The method involved searches in academic and legal databases (EUR-Lex, Curia, EU case law), as well as specialist journals. Official documents from international bodies such as the Council of Europe, OECD, UNESCO, the European Commission, EDPS/EDPB, as well as the latest European legislative acts. The criteria focused on primary sources and reviews published in peer-reviewed journals, policy and government reports, or scientific NGOs. The approach was mainly conceptual analytical, so principles and observations were extracted from various sources and compared to identify legal gaps and trends in neuroscience and/or behavioural science. The method adopted tends to be transdisciplinary, in the sense that it places itself between the legal-normative level, the technological level of design and algorithmic, persuasive architectures, digital interfaces, and the cognitive-ethical level of mental autonomy, freedom of thought, and psychological integrity. Transdisciplinary methodological insertions involved: (i) the use of concepts from neuroethics and cognitive sciences to interpret legal norms on data protection and prevention of manipulation; (ii) the integration of digital design and behavioural theories into the analysis of proportionality and risk assessment; (iii) correlating classical legal principles (dignity, autonomy, freedom of thought) with new forms of algorithmic influence. The analysis was carried out on three levels, starting with the descriptive level by mapping the applicable regulatory framework, continuing with the criticalevaluative level specifically designed to identify gaps in the protection of cognitive autonomy and the limitations of current DPIA or risk assessment mechanisms, and ending with a constructivenormative level for the formulation of proposals for innovative standards and mechanisms.

Based on this synthesis, a conceptual model called Authenticity-by-Design was developed, configured as a normative extension of the privacy by design paradigm, with operational principles articulated in decision-making stages. The model is schematically represented by a flowchart, designed to highlight the sequence of assessments, starting with the identification of cognitive risk, moving on to proportionality analysis, the implementation of technical and organisational measures, and ending with audit and review mechanisms.

We note that, as this is a synthesis study, the main limitations stem from the quality and diversity of the sources found, so that in some cases concepts such as mental integrity do not have a standard legal definition, which requires interpretations derived from context. However, the methodology aims for transparency and covers multiple disciplines.

### 1.2 Conceptual clarification of key terms

As the literature uses several closely related concepts, it is useful to clarify the terminology used in this article. Freedom of thought refers to the classical fundamental right recognized in international human rights law, which protects the internal formation of beliefs and opinions from external coercion. Mental integrity, a concept developed mainly in neuroethics and neurorights studies, focuses more specifically on the protection of the brain and neural processes from intrusive technological interventions. Mental self-determination refers to the ability of an individual to control and shape his or her own cognitive processes and decisions. In this article, the term cognitive autonomy is used as a general concept describing the ability of individuals to form judgments and make decisions independently in digital environments. Therefore, mental integrity and mental self-determination are suggested to be understood as specific dimensions of cognitive autonomy, while freedom of thought represents its broader foundation in terms of human rights.

## 2 Results

The Existing Legal Regime at the European Union and International Level.

### 2.1 EU and national law

The GDPR (EU Regulation 2016/679) regulates the protection of personal data, including sensitive categories of data, as presented at the beginning of this paper. In practice, data reflecting mental states or psychological characteristics may be treated as sensitive data. The GDPR sets out, in Article 25 and Recital 78, the principle of data protection by design and indicates that organisations must implement measures for the highest level of protection from the outset, such as data minimisation, encryption or pseudonymisation. However, the regulation does not explicitly mention the right to mental integrity or cognitive processes. As a result, the collection and processing of neural data (EEG signals) are largely regulated as ordinary personal data. Decisions such as consent to cookies are considered informed choices, but the GDPR does not provide for specific rules against manipulative interface design. Therefore, this regulation only covers personal data, leaving the psychological influence produced by interface elements (dark patterns that induce consent) unregulated. However, it is precisely this psychological influence that needs to be addressed in much greater detail and linked to the legal perspective (Johansen and Gregersen, 2024, p. 410).

In turn, the DSA (European Parliament and Council of the European Union, 2022) requires large internet platforms (VLOP, acronym for Very Large Online Platforms) to identify and mitigate the systemic risks of their services. The DSA explicitly mentions the mental health and well-being of users () and, in Articles 34–35, requires operators to assess the risks to them. Although mental integrity is not explicitly defined, researchers have noted that in the legal context of the DSA, it refers to the consumer’s right to control their mental states (Pałka and Ilczuk, 2026). A recent report explains that the DSA automatically protects three mental values: mental health, mental well-being and the right to mental integrity (Broughton Micova et al., 2024). However, the DSA only applies to large online platforms and does not include small websites, and implementation measures are still under development. Furthermore, restrictions on manipulative design are limited by the exclusion of practices already covered by other acts (UCPD, GDPR). At the level of EU Member States, for example in Romania, the educational document Artificial Intelligence & the Manipulation of Human Perception shows the national context of AI’s influence on perception and information manipulation, but does not present a primary national regulatory regime that goes beyond the EU legislative framework (Șcheau and Angheluș, 2024). They remain, in essence, national adaptations or implementations of EU rules.

The AI Act represents an important step in addressing technologically mediated manipulation. Article 5(1)(a) prohibits AI systems that deploy subliminal or deceptive techniques aimed at materially distorting behaviour, while Article 5(1)(b) protects vulnerable individuals from the exploitation of cognitive weaknesses (European Union, 2024, Regulation (EU) 2024/1689, Art. 5). The regulatory scope remains limited in two important respects. First, the prohibition applies only to AI systems, whereas many forms of behavioural influence in digital environments arise from interface architecture, persuasive design, or recommender systems that may not fall within the strict definition of AI. Second, the legal threshold of materially distorting behaviour may prove difficult to operationalise in practice, particularly in cases of subtle cognitive nudging that does not produce immediately demonstrable harm. As a result, a significant portion of everyday digital influence practices may remain outside the effective reach of the regulation.

Finally, we also mention here the DMA Regulation (European Union, 2022a, b), which imposes specific restrictions on large digital gatekeepers, such as Google and Meta in order to ensure fair competition. Articles 5(2) and 13 of the DMA require them not to use manipulative design patterns to prevent users from easily withdrawing their consent or being drawn into choices without a clear opt-out. In particular, Article 13 prohibits practices that, through technical or contractual means, circumvent the rules of the DMA, explicitly including dark patterns used to unfairly influence consumer decisions. However, the DMA only applies to designated dominant platforms and is limited to the services and behaviours included in its annexes; therefore, its cognitive protection restrictions are partial and do not cover all digital players or potential situations of manipulation (European Commission, Directorate General for Justice and Consumers, 2022).

With regard to other relevant acts, we note that at the European Union level, the relevant regulatory framework is the *Digital Services Act* (Regulation – EU – 2022/2065), part of the Digital Services Act Package, which is directly applicable in Member States. It was accompanied by resolutions and recommendations from the European Parliament, including the Resolution of 12 December 2023 on mental health, which emphasises the need to protect young people in the digital environment. The European Commission has launched a legislative initiative called *the Digital Fairness Act*, designed to address problematic practices in the digital environment, such as *dark patterns*, personalised marketing and other practices that affect consumers, as part of a broader reform of consumer law. At the time of writing this article, the proposal, which is under consultation and preparation, complements the current framework and highlights concerns about consumer protection in digital advertising (European Commission, 2025).

The Payment Services Directive (PSD2) and the European Union’s Data Act Regulation are normative documents that regulate transactions and access to data, even if they do not explicitly prohibit manipulative design techniques, instead providing the legal context for the protection of consumers and users in electronic transactions and access to data (Directive EU 2015/2366). Added to these is Regulation (EU) 2023/2854 (Data Act), which establishes harmonised rules on access to and use of data generated in the context of digital products and services, facilitating the exchange and use of data for the benefit of users and innovation. For example, the European Parliament resolution of 12 December 2023 on addictive design of online services and consumer protection in the EU single market (2023/2043 (INI) explicitly recognises that certain platforms use manipulative design and addictive interfaces, exploiting users’ psychological vulnerabilities and calls for legislative intervention. However, most of these initiatives are not fully legally binding and remain at the stage of proposals or guidelines.

## 3 Fundamental rights and case law

With regard to freedom of thought, we note that it is mentioned in general terms in Article 10 of the Charter of Fundamental Rights of the European Union (2012). The EU Charter of Fundamental Rights includes, in Articles 7 and 8, the right to privacy and data protection, as well as freedom of expression (Article 11). Although there is no specific article on freedom of thought in relation to online issues, experts (McCarthy-Jones et al.) argue that the right to freedom of conscience and thought should be extended to cover the protection of mental integrity. In the case law of the European Court of Human Rights (ECHR), Article 10 (freedom of thought, conscience and religion) and Article 7 (respect for private and family life) can be applied analogously as protection of the privacy of conscience, but there are no significant cases that explicitly sanction psychological manipulation by external means.

In the US, case law and doctrine on freedom of thought are more developed. The right to freedom of thought is an integral part of American constitutional thinking and has been analysed in doctrine and case law, even though there are not many direct cases concerning online manipulation. There are interpretations of the right to think and mental protection as an element of fundamental freedoms (Blitz, 2025). Specialist authors argue that freedom of thought is central to American jurisprudence on civil liberties and freedom of expression, particularly in interpretations of the First Amendment (Muñoz and Marinaro, 2025). In legal scholarship, freedom of thought is often treated as conceptually distinct from freedom of expression and has increasingly been discussed as a possible doctrinal foundation for the protection of cognitive liberty, especially in the context of emerging risks of mental manipulation through digital technologies (Ienca and Vayena, 2019).

An interesting comparative development can be observed in Chile. In the case *Girardi v. Emotiv*, the Supreme Court of Chile (9 August 2023) addressed the protection of neurodata collected through an EEG device marketed by Emotiv Inc. This decision is widely regarded as the first judicial case dealing directly with neuroprivacy. The Court ordered the deletion of the collected brain data and affirmed that such data may affect constitutional rights to physical and psychological integrity as well as privacy (Cornejo-Plaza et al., 2024). Chile has also taken a further step at the constitutional level. Through constitutional reform, the concept of mental integrity was explicitly introduced as a fundamental right, reflecting the growing recognition of so-called neurorights in legal systems (Ley 21,383, 2021; Do et al., 2024). In contrast, the European Union has so far adopted a more indirect approach, addressing risks to cognitive autonomy primarily through sectoral regulation such as data protection law (GDPR), platform governance (DSA) and artificial intelligence regulation (AI Act).

This comparison suggests complementary lessons from which we understand that the Chilean model demonstrates the potential advantages of explicit constitutional recognition of mental integrity as a fundamental right, while the EU approach offers a more detailed regulatory framework capable of addressing specific technological practices. A dialogue between these approaches may therefore contribute to the development of more comprehensive safeguards for cognitive autonomy in the digital environment.

Working groups and bodies such as the OECD, UNESCO and the Council of Europe are proposing new formulations. The OECD defines cognitive liberty as the right to mental self-determination [Organisation for Economic Co-operation and Development (OECD), 2019]. A 2024 CoE report even recommends the explicit inclusion of mental integrity and cognitive liberty in current human rights treaties (Bertoni and Ienca, 2024, p. 12). Although these proposals are not yet law, they highlight the development in legal discourse of the need to specifically recognise the mental and cognitive domain as worthy of special protection.

## 4 Empirical studies and evidence of cognitive manipulation

The literature in neuroscience and behavioural economics shows multiple scenarios and mechanisms through which digital media can influence the mind. Recent empirical studies associate heavy use of social media with diminished cognitive abilities and increased mental health problems (Hunt et al., 2018). For example, research from 2023 indicates a correlation between excessive social media consumption, decreased attention, and increased risk of anxiety and depression in users (Keles et al., 2019). Other experiments demonstrate the subtle effects of interface design, such as changing the colour or text of buttons, which can alter user choices without them realising (Mildner et al., 2023, p. 8). The EDPB guidelines for digital businesses classify dark patterns that lead users to unintended and unwanted decisions, affecting their ability to make conscious decisions about their data [European Data Protection Board, 2022, Executive Summary, para. 2]. Furthermore, experiments in psychology and behavioural economics show that many digital nudges rely on universal cognitive biases such as the status quo effect, anchoring, and aversion to loss, which are exploited by designers to increase click-through or purchase rates (Cross, 2001).

Neuromarketing data and market studies also indicate a long-term impact in the sense that people who are constantly exposed to digital persuasion strategies may experience changes in their preferences and level of autonomy. For example, analysis of recommendation systems shows the risk of creating filter bubbles (closed information communities) that undermines the ability to make decisions based on complete information (Car and Cassetti, 2025). In summary, we can say that the evidence suggests that users risk being subtly manipulated by attributing behaviour to searches or likes, without this being detected by traditional legal mechanisms. However, from a legal perspective, this influence does not automatically constitute visible harm, but is a systemic risk that is difficult to quantify statistically. Thus, the threshold of evidence required for direct legal intervention may be difficult to reach, highlighting the need for preventive measures and tools for assessing cognitivebehavioural risks (Istace, 2023, p. 220) (Table 1).

**Table 1:** Comparison of the main relevant European/legislative instruments for the protection of cognitive autonomy (key elements vs. gaps, jointly created by the authors).

Instruments/regulatory framework	Scope of application	Formal cognitive protection	Main gaps regarding cognitive autonomy
GDPR (European Union, 2016/679)	Personal data	Protects sensitive data (health, biometrics); requires confidentiality and minimisation	Does not mention thought control; nudging interfaces (implicit defaults) are not targeted; formal consent (Art. 7) can be influenced by UI.
DSA (European Union, 2022a, b)	Online platforms (VLOP)	Imposes risks to mental health and well-being	Only applies to large platforms; concept of mental integrity is vague; only risk assessment, no strict technical standards.
AI Act (European Union, 2023)	AI systems	Prohibits AI that uses subliminal or manipulative techniques	Applies only to AI; does not cover other technologies; implementation period (until 2026); broad definitions may leave room for interpretation.
DMA (European Union, 2022a, b)	Gatekeepers (Big Tech)	Prohibits manipulative choice design (Art. 13); favours withdrawal of consent	Only dominant platforms; limits on services and practices included; does not directly protect mental integrity; primarily targets consent, not general psychological distress.
Data Act (proposed)	Data exchange between parties	Protects consumers from interface techniques that deceive users and undermine their autonomy	Focuses on data transactions between SMEs and platforms; does not cover all behavioural aspects of UX; still in the process of being adopted.
Instruments/Regulatory framework	Scope of application	Formal cognitive protection	Main gaps regarding cognitive autonomy
UCPD Directives (EU)	B2C commercial practices	Prohibits aggressive and misleading commercial practices; includes some examples of dark patterns in the annex	Generic terms, does not mention advanced digital concepts; applicable only to commercial transactions; risk of inconsistency in application.
EU Charter of Fundamental Rights	General fundamental rights	Art. 7/8 protects privacy and data; Art. 10 freedom of thought	No specific article on freedom of thought in the digital environment; requires broad interpretation to cover cognitive threats.
Human Rights Convention	Fundamental rights (ECHR)	Art. 9 (thought/religion), Art. 8 (privacy)	Does not refer to modern technological influences; limited interpretation of the private sphere of thought.
OECD/CoE Recommendations	International policies	Promotes neurorights: mental integrity, cognitive freedom	Not legally binding; serves as a model, does not impose direct sanctions.
Professional frameworks (EDPB, EDPB, etc.)	Voluntary guidelines and codes	Guidelines on dark patterns and cookie consent (GDPR)	Non-binding recommendations; applicability limited to GDPR issues.

Additional empirical evidence further confirms the behavioural effects of digital interface architecture and algorithmic curation. Controlled experimental studies on so-called dark patterns show that manipulative interface designs significantly influence user decision-making. For instance, Mathur et al. (2019) identified more than 1,800 instances of dark patterns across over 11,000 e-commerce websites and demonstrated that such design practices systematically steer users toward decisions favourable to service providers. On the same line, laboratory experiments conducted by Luguri and Strahilevitz (2021) indicate that manipulative choice architectures can substantially increase the likelihood that users consent to data-sharing practices or subscription services they would otherwise decline. Research on recommender systems also highlights the behavioural consequences of algorithmic personalisation. Studies analysing large-scale social media data suggest that algorithmic filtering can reinforce informational homogeneity and contribute to the formation of filter bubbles, thereby limiting users’ exposure to diverse viewpoints and potentially affecting democratic deliberation (Flaxman et al., 2016).

Taken together, these empirical findings indicate that digital environments can shape cognitive processes not only through overt persuasion but also through subtle interface architectures and algorithmic curation mechanisms. Such influence operates cumulatively and often remains invisible to users, which makes it particularly difficult to detect within traditional legal frameworks that rely on identifiable and immediate harm. This reinforces the argument that regulatory approaches should incorporate preventive mechanisms capable of assessing cognitive-behavioural risks before significant harm materialises.

### 4.1 Scientific evaluation and case studies

There are still no quantitative legal studies that directly demonstrate the influence of algorithms on users’ decisions in litigation contexts, but the experimental literature and case studies from the technology sector provide some important empirical indications. Research shows that algorithmic recommendations on social networks run the risk of amplifying cognitive biases and shaping patterns of information exposure (Flaxman et al., 2016). Policy reports and investigations into the practices of digital platforms indicate that insufficient regulatory oversight allows the exploitation of behavioural vulnerabilities in online environments (European Commission, 2021). In addition, journalistic investigations related to the Cambridge Analytica scandal revealed how large-scale profiling of personal data was used to generate highly personalized political messages designed to influence electoral behaviour (Cadwalladr and Graham-Harrison, 2018). Although such practices are not legally sanctioned in many jurisdictions, they present the potential risks associated with the exploitation of behavioural and psychological data.

Choice studies demonstrate that implicit choices strongly affect user behaviour, as individuals tend to follow predefined selections rather than actively alter them (Johnson and Goldstein, 2003). Viewed through this logical lens, empirical findings suggest that protecting cognitive autonomy should rely on both reactive legal mechanisms and preventive regulatory and design approaches that anticipate subtle behavioural influences before measurable harm occurs.

## 5 Legislative gaps

The comparative analysis highlights a fragmented and incomplete framework in the EU. Although manipulative practices have been addressed in various directives and regulations, there is no single legal definition of psychological manipulation. The current regime is fragmented and lacks clear definitions, leading to uncertainty in application (Car and Cassetti, 2025). European acts mention dark patterns only in specific contexts, such as the GDPR on consent, the DSA on platforms, the UCPD on commercial transactions, and the AI Act on AI. Thus, cognitive autonomy does not have a uniform legal benchmark. If we look at the example of an interface designed to trick a user into signing an online contract, it may violate the UCPD or DSA, but if the same type of nudging is used to influence news consumption, there is no dedicated rule. Furthermore, the exclusions from the DSA for things already regulated and the secondary role of the GDPR (data only) mean that any new situation could escape legislative control (European Commission, 2026). Current privacy by design requirements are difficult to assess externally, and we see how, for example, the GDPR requires protection by design but does not impose cognitive design audits for interfaces. In the absence of precise standards, many potential abuses go unpunished. This situation has been criticised by analysts who point out that rational humans as consumers do not always reflect real behaviour in the digital space, creating a regulatory vacuum regarding persuasive techniques (European Commission, Directorate General for Justice and Consumers, 2022).

## 6 Proposed ‘Authenticity-by-Design’ framework

Based on the gaps identified, we propose a set of principles and concrete measures for Authenticity-by-Design, analogous to the concept of privacy by design, but focused on cognitive integrity:

The first of these refers to respect for mental autonomy. Digital systems must support cognitive braking options through additional steps before important decisions and provide real options for informed consent and withdrawal. User consent should be given in conditions of maximum transparency. According to a Council of Europe report, a core component of cognitive freedom is the right to withdraw from the collection of neural data at any time (Bertoni and Ienca, 2024). By analogy, digital environments should take whatever measures are necessary so that users can opt out of interface features that rely on behavioural influence mechanisms. In practice, this does not require users to independently detect all forms of cognitive influence. Rather, platforms should provide clear indicators when personalisation, recommendation algorithms or behavioural push mechanisms are active, together with easily accessible options allowing users to disable such features or revert to neutral interface settings. Establishing transparency and opt-out mechanisms would operationalise the principle of cognitive autonomy in digital design, while preserving the user’s ability to make informed and voluntary choices.

In terms of transparency and explanations, algorithms that can influence behaviour must be descriptive and verifiable. Platform operators should explain, in accessible terms, how recommendations can influence users’ attention and emotions. This algorithmic transparency increases trust and allows for external auditing.

Psychic privacy must be placed at the centre of attention so that users’ neural data and mental signatures are treated as enhanced protection data. Legislation should recognise specific rights over mental data, drawing inspiration from proposals made so far that have presented the protection of mental integrity as an evolution of the right to privacy. Thus, any capture or analysis of data about cognitive states, such as emotions detected by AI, would require explicit consent and special security measures.

Another principle would be authentic user-oriented design. Interfaces should be designed to help users express their real needs and preferences. In practice, companies should be encouraged to adopt cognitive impact assessments (MPIA – Mental Privacy Impact Assessment, inspired by existing Privacy Impact Assessments) before launching products, similar to DPIA in the GDPR. MPIA would assess the risks of psychological manipulation and suggest mitigation measures. Concrete components could include introducing cognitive friction through reflection steps before final clicks or reducing excessive visual stimuli, especially for vulnerable groups, such as the elderly and minors. In practice, the assessment would be carried out mainly by the service provider before the implementation of digital systems with a significant risk of behavioural influence or cognitive manipulation. The documentation produced through the MPIA could be subject to review by existing supervisory authorities, such as national data protection authorities or digital service coordinators under the Digital Services Act.

Alternatively, these bodies could require additional safeguards or corrective measures if cognitive risks are identified. As cognitive harm is often difficult to quantify in traditional judicial proceedings, enforcement would likely rely on a preventive regulatory approach. One possible mechanism would be to recognise relative presumptions of risk for certain manipulative interface practices such as dark patterns or covert behavioural nudging techniques. In such situations, the burden of proof would be partly shifted to service providers, requiring them to demonstrate that their design choices do not undermine the cognitive autonomy of users. The solution allows regulators and courts to address systemic risks of cognitive manipulation without requiring individuals to prove complex psychological harm in each case.

Last but not least, education and awareness must be a priority here too. Users must be actively informed about potential manipulation and the use of their mental data. Awareness campaigns have the power to explain the difference between brain data and other personal data, increasing the ability to review consent. The involvement of civil society, academia and other stakeholders is essential to ensure the accountability of technology providers.

**Figure 1:**
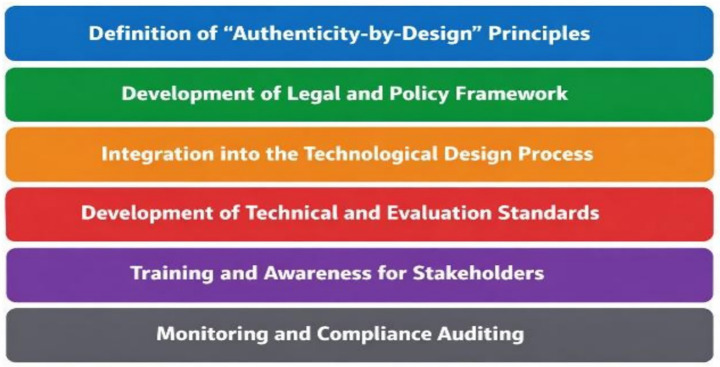
Summarises the key steps for implementing the proposed framework.

The diagram, developed by the authors, summarises the key steps for implementing this framework (Figure 1).

At the operational level, the measures refer to the revision of existing EU legislation. Why not extend the scope of the DSA to include algorithmic manipulation as a prohibited practice, or by requiring that any dark pattern be evaluated and approved by the authorities. In the technical field, it would be useful to publish mandatory guidelines on ethical design as UI standards that eliminate intimidation or fear-inducing tactics. The new principles should also be transposed into certification criteria for digital products and public procurement criteria for educational software without harmful mechanisms, for example.

## 7 Discussions and conclusions

The application of Authenticity-by-Design principles would have positive effects primarily because, at the societal level, it would strengthen democracy by protecting citizens’ ability to think independently. At the same time, at the public health level, it would contribute to reducing the risks of addiction and anxiety induced by technology. It would also generate new, more user-friendly technologies, such as platforms that recommend content based on criteria of authentic relevance rather than immediate profit. In the legal arena, this framework would require cooperation between institutions such as parliament, data protection authorities, or standardisation and industry bodies.

The new cognitive standards must be adopted by certification bodies (similar to ISO27001 for security), but extending the scope of verification to psychological components.

The limitations of the current work include inherent conceptual uncertainties. The notion of cognitive autonomy does not yet have a clear definition in law, which makes it difficult to draft precise texts. Similarly, the implementation of the proposed measures is likely to encounter technical and economic resistance, as tech companies could argue that any additional restrictions reduce their competitive advantage or make it difficult to innovate without a harmonised international standard. On the scientific front, there is still no y consensus on cognitive impact measures, an issue that needs to be resolved in the near future. For the time being, no one has defined and approved a single method for measuring psychological influence in digital interfaces.

Future research should address these points through interdisciplinary experiments to establish tools for assessing digital manipulation; socio-legal studies on the effectiveness of existing regulations, such as the hypothesis that the DSA’s ban on dark patterns truly reduces manipulative behaviour; and the development of metrics for mental integrity and cognitive distance. On the other hand, there is a need for extensive public dialogue, as McCarthy-Jones also recommends, because society must decide how to balance the risk of technologies with the need for mental autonomy.

Despite the uncertainties, it is clear that the current situation requires proactive action. Fundamental rights already refer, in principle, to the mind remaining untouched if we consider freedom of thought, which has absolute protection under international norms. As we have emphasised, a person must be able to withdraw their neural data and exercise voluntary control over mental information. Regulatory proposals in recent months recognise this at a conceptual level. The next step is to transform them into operational legal instruments, and this report provides a starting point through the principles and steps suggested.

It can be concluded that protecting cognitive autonomy in the digital space is indispensable for human dignity and social cohesion. Although the difficulties are considerable when it comes to legal definition, coordinated regulation, or scientific impact assessment, there is already a foundation on which effective policies can be built. The Authenticity-by-Design approach proposed here draws on legal, scientific, and ethical perspectives and ultimately proposes a coherent plan of action. For the desired effects, decision-makers must consider these recommendations and collaborate inter-, multi-, and transdisciplinary with specialists in neuroscience and ethics to transform theoretical cognitive rights into protected realities.
